# Association between Low Protein Intake and Mortality in Patients with Type 2 Diabetes

**DOI:** 10.3390/nu12061629

**Published:** 2020-06-01

**Authors:** Takuya Yamaoka, Atsushi Araki, Yoshiaki Tamura, Shiro Tanaka, Kazuya Fujihara, Chika Horikawa, Rei Aida, Chiemi Kamada, Yukio Yoshimura, Tatsumi Moriya, Yasuo Ohashi, Yasuo Akanuma, Hideki Ito, Hirohito Sone

**Affiliations:** 1 Department of Diabetes, Metabolism and Endocrinology, Tokyo Metropolitan Geriatric Hospital, 35-2 Sakaecho, Itabashi-ku, Tokyo 173-0015, Japan; tak62923@gmail.com (T.Y.); tamurayo@tmghig.jp (Y.T.); hideki_ito@tmghig.jp (H.I.); 2 Department of Clinical Biostatistics, Graduate School of Medicine, Kyoto University, Yoshida-Konoe-cho, Sakyo-ku, Kyoto 606-8501, Japan; tanaka.shiro.8n@kyoto-u.ac.jp (S.T.); aida.rei.5c@kyoto-u.ac.jp (R.A.); 3 Department of Hematology, Endocrinology, and Metabolism, Niigata University Faculty of Medicine, 1-757 Asahimachi-dori, Chuoh-ku, Niigata 951-8510, Japan; kafujihara-dm@umin.ac.jp (K.F.); horikawa@unii.ac.jp (C.H.); sone@med.niigata-u.ac.jp (H.S.); 4 Department of Health and Nutrition, University of Niigata Prefecture Faculty of Human Life Studies, Niigata 950-8680, Japan; 5 Training Department of Administrative Dietitians, Shikoku University, 123-1 Ebisuno, Furukawa, Ojin-cho, Tokushima, Tokushima 771-1151, Japan; c-kamada@shikoku-u.ac.jp (C.K.); yyoshimura@shikoku-u.ac.jp (Y.Y.); 6 Health Care Center, Kitasato University, Sagamihara, Kanagawa 252-0373, Japan; moriy@kitasato-u.ac.jp; 7 Department of Integrated Science and Engineering of Sustainable Society, Chuo University, 1-13-27 Kasuga, Bunkyo-ku, Tokyo 112-8551, Japan; ohashi@epistat.m.u-tokyo.ac.jp; 8 The Institute for Adult Diseases, Asahi Life Foundation, 2-2-6 Nihonbashibakurocho, Chuo-ku, Tokyo 103-0002, Japan; y-akanuma@asahi-life.or.jp

**Keywords:** diabetes, mortality, protein intake, nutritional support, aging

## Abstract

The aim of this study was to investigate the association between protein intake and mortality risk in patients with type 2 diabetes. We analyzed a pooled data of 2494 diabetic patients from two prospective longitudinal studies. Nutritional intake was assessed using a Food Frequency Questionnaire at baseline. Protein intake per body weight (kg) per day was categorized into quartile groups. Adjusted hazard ratios (HRs) and 95% confidence interval (CI) were calculated using Cox regression analysis. During the six-year follow-up, there were 152 incidents of all-cause mortality. The HR for mortality in the lowest quartile of protein intake per body weight compared with the highest quartile was 2.26 (95% CI: 1.34–3.82, *p* = 0.002) after adjustment for covariates. Subgroup analyses revealed significant associations between low protein intake and mortality in patients aged over 75 years or under 65 years. After further adjustment of the total energy intake, a significant association between protein intake and mortality remained in patients aged ≥ 75 years, whereas the association was attenuated in those aged < 65 years. Our results suggest that adequate protein intake is necessary in older diabetic patients over 75 years, whereas with diabetes, whereas whole optimal total energy intake is required in younger patients with type 2 diabetes.

## 1. Introduction

Diabetes is associated with a high risk of mortality despite recent advances in treatment [1]. Hyperglycemia, hypoglycemia, hypertension, dyslipidemia, and obesity, all of which could be causes of cardiovascular complications, may explain the increased risk of death in diabetes, but the exact cause is unknown. In older adults with diabetes, malnutrition (low body mass index (BMI)), sarcopenia, and frailty have been reported to be risk factors for mortality [2,3,4].

Although diet therapy is a basal therapy for all patients with type 2 diabetes, it is unknown how macronutrients or other nutritional components affect mortality in persons with diabetes. Increased protein intake in individuals with diabetes can exacerbate nephropathy, which could lead to death. Conversely, reduced protein intake in patients with diabetes, especially in older people, might increase the risk of death by causing sarcopenia and frailty. Studies on the effects of protein restriction on renal function in patients with diabetes showed conflicting results on changes in estimated glomerular filtration rate (eGFR) or incident end-stage renal disease [5,6,7] and increased mortality [8].

We aimed to investigate the association between protein intake and the risk of mortality in Japanese patients with type 2 diabetes from the analysis of pooled data from two prospective longitudinal studies, namely the Japan Diabetes Complications Study (JDCS) and the Japanese Elderly Diabetes Intervention Trial (J-EDIT).

## 2. Materials and Methods

### 2.1. Study Populations

This study was a pooled analysis of two cohorts, namely JDCS [9,10] and J-EDIT [11]. In the JDCS, 2033 Japanese patients with type 2 diabetes aged 40–70 years, whose hemoglobin A1c (HbA1c) levels were ≥7.0%, were recruited between January 1995 and March 1996 and were randomized into a conventional treatment group and a lifestyle intervention group. The median follow-up time was 7.8 years [9,10]. The J-EDIT was another randomized controlled trial of intensive and conventional treatments for diabetes. In the study, 1173 Japanese aged 65–85 years with type 2 diabetes, whose HbA1c levels were ≥7.9% or ≥7.4%, with at least one abnormal condition of body mass index (BMI), blood pressure, or lipids, were enrolled between March 2001 and February 2002. Patients who had recent history (i.e., within 6 months) of myocardial infarction, stroke, cancer, acute or serious illness, aphasia, and severe dementia were excluded from the study. Follow-up time was 6 years, and the dropout rate after 6 years was 8.9% (104 cases) [11]. Diabetes mellitus was diagnosed according to the “Report of the Committee of the Japan Diabetes Society on the Classification and Diagnostic Criteria of Diabetes Mellitus”, which is almost identical to those of the WHO, in terms of thresholds for glucose levels.

The JDCS and J-EDIT received approval from the ethical committees of all participating institutes, and written informed consent was obtained from all patients before enrollment.

In the JDCS and J-EDIT, almost similar information about anthropometric and laboratory tests, other clinical variables, and outcomes for each patient were collected at a central data center through an annual report from each investigator. Details of the data in JDCS and J-EDIT were previously published [9,11]. The pooled data of a total of 2494 patients with type 2 diabetes who received nutritional assessment at baseline were analyzed.

Alcohol consumption and smoking status (with or without alcohol consumption, and current smoker or non-smoker) were determined using self-reported questionnaires [12]. Physical activity (with or without exercise habits) at baseline was assessed using self-administered questionnaires. The patients reported their average frequency (times per week) and duration (minutes per day) of normal walking, brisk walking, jogging, golfing, tennis, swimming, aerobics dancing, cycling, and other exercises.

### 2.2. Laboratory Tests

Patients were assessed yearly after the baseline evaluation. Mean values for at least two measurements each year were obtained for HbA1c, fasting plasma glucose, and fasting serum lipids. HbA1c assays were performed according to procedures outlined by the Laboratory Test Committee of the Japan Diabetes Society (JDS), which were converted using the following formula: HbA1c (JDS) (%) = 0.98 × HbA1c (National Glycohemoglobin Standardization Program) (%)-0.25%. All other laboratory tests were performed at each participating institute. Serum low-density lipoprotein (LDL)-cholesterol was calculated using the Friedewald equation, except where triacylglycerols exceeded 4.52 mmol/L (400 mg/dL), in which case LDL cholesterol data were treated as “missing”. This was applicable to 19 participants. All other measurements, including those for body weight, blood pressure, and 12-lead electrocardiography, were performed at least once yearly.

Estimated glomerular filtration rate (eGFR) was calculated according to the Japanese coefficient-modified Modification of Diet in Renal Disease study equation [13]: eGFR (mL/min/1.73 m^2^)  =  194 × (serum creatinine)-1.094 × (age) ^−0.287^ (× 0.739, when female). The urine albumin-to-creatinine ratio (urine albumin excretion ratio) was measured and calculated on the basis of 1 g of urinary creatinine.

### 2.3. Nutritional Assessment

The Food Frequency Questionnaire based on food groups (FFQg) was used to gather information about nutritional and food intake and was administered at baseline [14]. This 46-item questionnaire describes a standard portion size for each food item, and each patient was asked to select the portion size that they typically consume (50%, 100%, or 200% of a standard portion) and the intake frequency per week for each food. We calculated the intakes of total energy, nutrients, and food groups using standardized software for population-based surveys and nutritional counseling in Japan (Excel EIYO-Kun, version 4.5; Shikoku University Nutritional Database, Kenpakusha, Japan). External validation of the FFQg was made through comparison with dietary records kept for 7 days by 66 individuals aged 19–60 years [15]. The ratios of the estimates obtained by the FFQg against those by the dietary records were 104% on average and ranged from 72% to 121%.

### 2.4. Outcome Measures

The endpoint was all-cause mortality. Information regarding vital status and causes of death were obtained through an annual report form that included detailed findings at the time of the event from each participating diabetologist who was providing care to those patients. Causes of death were classified based on the ninth revision of the International Classification of Diseases (ICD-9) Clinical modification codes for cardiovascular disease (diagnosis codes 390–452), cancer (diagnosis codes 140–208), and other miscellaneous causes.

A fatal or first non-fatal manifestation of coronary heart diseases (CHDs) (angina pectoris or myocardial infarction) was diagnosed according to criteria defined by the Multinational Monitoring of Trends and Determinants in Cardiovascular Disease (WHO/MONICA) project [16,17], as previously described [9,11]. Stroke was defined as clinical signs of a focal neurological deficit with rapid onset that lasts > 24 h, confirmed by the findings of either brain CT or MR imaging [18,19]. No cases of asymptomatic lesions detected using brain imaging (i.e., silent infarction) were included. The endpoints were adjudicated by central committees comprising experts in diabetology, as well as cardiology, who were masked to risk factor status, including information on physical activity, and was based on additional data, such as a detailed history, sequential changes in electrocardiogram and serum cardiac biomarkers, and results of coronary angiography. Information on other clinical variables for each individual was also collected through the annual report.

### 2.5. Statistical Analysis

The characteristics of the JDCS and J-EDIT participants were summarized as mean ± standard deviation (SD). After protein intake per body weight (kg) per day was categorized into quartile groups (Q1: 0.34 (<0.92 g/kg/day; Q2: 0.92–1.15 g/kg/day; Q3: 1.15–1.41 g/kg/day; and Q4 (reference): >1.41 g/kg/day), hazard ratios (HRs) for all-cause mortality were estimated using Cox regression analysis. Analyses were performed on the following models: model 1 (crude model), model 2 (adjusted for age, sex, BMI, HbA1c, systolic blood pressure (SBP), LDL cholesterol, smoking, and alcohol intake), model 3 (model 2 + urine albumin creatinine ratio (UACR), eGFR, and exercise), model 4 (model 3 + energy intake), and model 5 (model 3 + carbohydrate intake). Subgroup analyses in the Cox regression analysis were performed using the following groups: sex (men vs. women), age (<65 years, 65–74 years, ≥75 years), HbA1c (<7.5% vs. ≥7.5%), SBP (<135 mmHg vs. ≥135 mmHg), and total energy intake (<1533, 1533–1833, ≥1834 kcal/day). Patients with missing data in the analyses were excluded (complete case analysis). All *p*-values were two-sided, and *p* < 0.05 was considered to indicate statistical significance. Data management was conducted at a central data center. Statistical analysis was performed at another center using SPSS v26 (IBM Corp., Armonk, NY, USA).

## 3. Results

Baseline characteristics of patients by quartiles of protein intake per body weight are presented in Table 1. Protein intake per actual body weight (kg) per day was categorized into quartile groups: Q1 at 0.34–0.92 g/kg/day (*n* = 624), Q2 at 0.92–1.15 g/kg/day (*n* = 623), Q3 at 1.15–1.41 g/kg/day (*n* = 624), and Q4 at 1.41–3.79 g/kg/day (*n* = 623). Data on a total of 2494 out of 3028 patients were used for analysis, after patients with missing data on protein intake were excluded. For dietary intake, mean protein intake ranged from 0.8 to 1.7 g/kg/day across quartiles. Mean energy intake across quartiles ranged from 1411 to 2072 kcal/day. Age, HbA1c, exercise, urine albumin-to-creatinine ratio, and eGFR did not differ significantly according to quartiles of protein intake.

During the follow-up period (mean: 6.1 ± 2.5 years), there were 152 incidents of all-cause mortality in the crude model. They included 57 cancers, 23 cardiovascular diseases, 16 sudden deaths, 31 others, and 25 unknown causes. The follow-up rate in model 1 was 96.6% (2408/2494) for all-cause mortality. In the confounder-adjusted analysis (model 4), there were 135 incidents.

**Figure 1:**
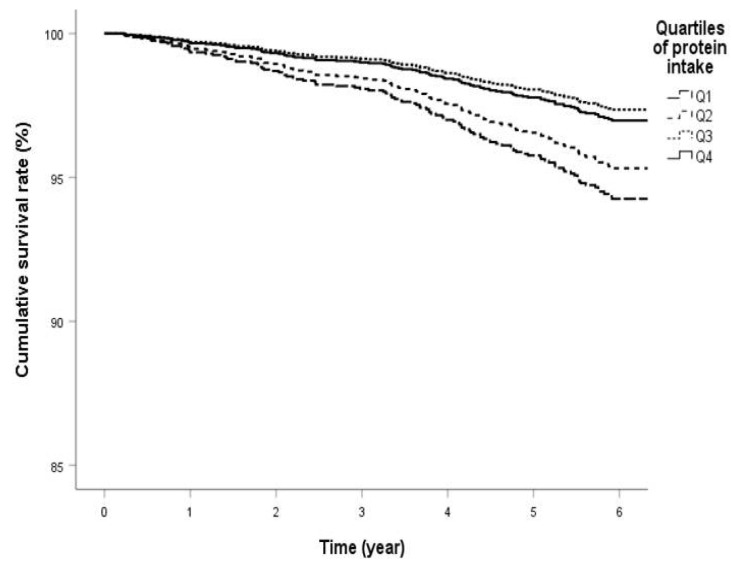
Survival curve of quartile groups by protein intake in patients with diabetes mellitus.

Table 2 shows HRs for protein intake estimated using the Cox regression models. In the categorized groups, the highest cumulative survival rate was observed in the Q3 group (1.15–1.41 g/kg/day), followed by the Q4 group (1.41–3.79 g/kg/day), Q2 group (0.92–1.15 g/kg/day), and Q1 group (0.34–0.92 g/kg/day) (Figure 1). The analyses in models 1, 2, and 3 showed a significant association between reduced protein intake and all-cause mortality. The HR for mortality in the lowest quartile of protein intake compared with the highest quartile was 2.26 (95% CI: 1.34–3.82, *p* = 0.002) in model 3. However, in the analysis in model 4, which was adjusted for covariates, including energy intake, the association between protein intake and all-cause mortality attenuated.

Q1 protein intake ranged from 0.34 (minimum observed level) to 0.92 g/kg/day. Q2 protein intake ranged from 0.92 to 1.15 g/kg/day. Q3 protein intake ranged from 1.15 to 1.41 g/kg/day. Q4 protein intake ranged from 1.41 to 3.79 (maximum observed level) g/kg/day. Cumulative survival rate of the quartile groups is shown in the Cox regression analysis on model 4 adjusted for age, sex, BMI, HbA1c, SBP, LDL cholesterol, smoking, alcohol intake, UACR, eGFR, exercise, and energy intake. Q1 is the lowest cumulative survival rate, and Q3 is the highest. 

**Figure 2:**
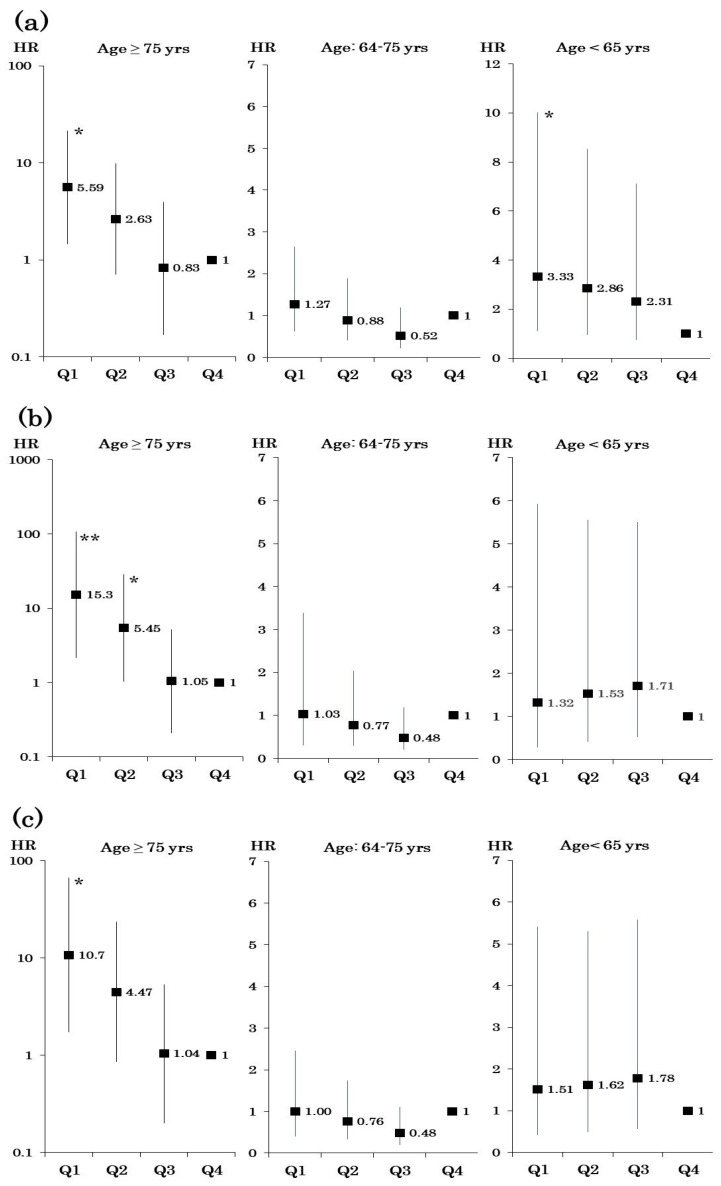
(**a**) Association between protein intake and all-cause mortality by age groups after adjustment for age, sex, body mass index (BMI), hemoglobin A1c (HbA1c), systolic blood pressure (SBP), low-density lipoprotein (LDL) cholesterol, smoking, alcohol intake, urine albumin creatinine ratio (UACR), estimated glomerular filtration rate (eGFR), and exercise (model 3). (**b**) Association between protein intake and all-cause mortality by age groups after further adjustment for total energy intake (model 4).(**c**) Association between protein intake and all-cause mortality by age groups after further adjustment for carbohydrate intake (model 5). *p <0.05, **p <0.01 vs Q4.

Subgroup analyses according to sex, age, HbA1c, SBP, total energy intake, eGFR, and duration of diabetes are shown in Table 3. Significant associations between protein intake and mortality were observed in patients aged ≥ 75 years or < 65 years, with HbA1c ≥ 7.5%, or SBP ≥ 135 mmHg, even after adjustment for covariates, including albuminuria, renal function, and exercise (model 3). In the group aged ≥ 75 years, significant associations between low protein intake and mortality were observed in all four models (the result of model 3 is shown in Figure 2a). The HR for all-cause mortality after further adjustment for total energy intake for the lowest quartile of protein intake compared with the highest quartile was 15.3 (95% CI: 2.17–107.3) (model 4, Figure 2b). After adjustment for carbohydrate intake instead of total energy intake, the risk of mortality in the lowest quartile group remained significant (model 5, Figure 2c). In contrast, in the young group aged < 65 years, the significant association between low protein intake and mortality disappeared after adjusting for total energy intake (model 4, Figure 2b) or carbohydrate intake (model 5, Figure 2c). The intake of saturated fat or salt did not affect the association between protein intake and mortality in total and subgroup analysis of age (data not shown).

When animal proteins (defined as consumption of seafood, meat, egg, and milk) and vegetable proteins (defined as consumption of legume, green and other vegetables) were examined separately, the association between decreased intake of vegetable proteins and death was particularly remarkable (Table 4). No significant association was found between excess intake of animal protein and death.

**Figure 3:**
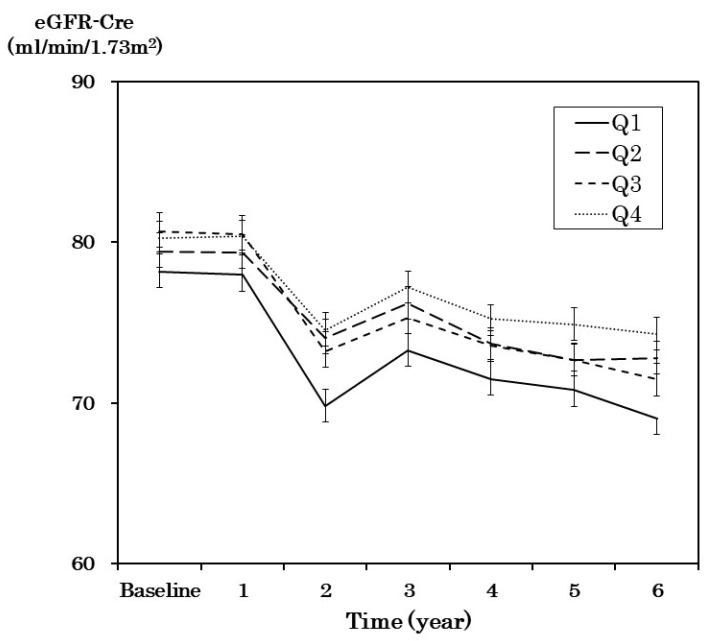
Changes in eGFR-creatinine (eGFR-Cre) in the quartile group of protein intake during the 6-year follow-up period.

Figure 3 shows the change in eGFR between groups categorized by protein intake in the six-year follow-up period. Changes in eGFR were defined as the difference in eGFR during follow-up and eGFR at baseline. Between the groups, baseline eGFR and the sequential changes in eGFR were not significantly different after one-way analysis of variance. We performed multiple linear regression analysis for the changes in eGFR using the following factors, namely age, sex, HbA1c, systolic blood pressure, UACR, and quartiles of protein intake per body weight. Changes in eGFR were similar in the quartile groups of protein intake per body weight.

Mean eGFR-Cre (mL/min/1.73 m^2^) and SE of quartile groups were plotted at baseline and the following six years. The protein intake was 0.34–0.92 g/kg/day in the Q1 group, 0.92–1.15 g/kg/day in the Q2 group, 1.15–1.41 g/kg/day in the Q3 group, and 1.41–3.79 g/kg/day in the Q4 group.

## 4. Discussion

In the pooled analysis of two Japanese cohorts, low protein intake was associated with increased mortality in patients with type 2 diabetes mellitus. Significant associations between protein intake and mortality were found, especially in patients aged > 75 years or < 65 years, those with ≥HbA1c 7.5%, or SBP ≥ 135 mmHg, and remained after adjustment for covariates, including albuminuria and renal function. To our knowledge, this is the first study to clarify the association between reduced protein intake and death in patients with diabetes.

In our study, the association between reduced protein intake and mortality varied with age. When total energy and exercise were corrected, the mortality rate increased as the protein intake decreased in the group with patients aged > 75 years. In contrast, in the group with patients aged < 65 years, the significant association disappeared. In the youngest group, low total energy intake or carbohydrate intake, rather than protein intake, may have affected mortality. The differential effects of protein intake on mortality according to age were observed in other reports for the general population. In people aged > 65 years, the risk of mortality decreased as protein intake increased, whereas risk of mortality increased as protein intake increased in those aged < 65 years [20]. As a mechanism, high protein intake may cause excessive secretion of insulin-like growth factor-I (IGF-I) in people aged < 65 years to cause cancer and death, although clear differences in cause of death between the age groups were not observed, due to the small sample size.

In patients aged ≥ 75 years, the association between low protein intake and mortality persisted, even after adjusting for total energy intake. Low protein intake is associated with sarcopenia and frailty in the diabetic and general populations, respectively [21,22], which may lead to increased risk of disability and death in older people with diabetes [4,23]. Furthermore, protein–energy malnutrition is a feature of malnutrition among older people [24]. Older patients with diabetes mellitus are likely to have sarcopenia, frailty, and malnutrition [25]. Although we could not perform detailed analyses on the relation between protein intake and cause-specific mortality because of a small sample size, the decrease in protein intake in older people will likely increase susceptibility to infection, due to decreased immunity and death from infection. In fact, malnutrition is known to be a significant risk factor for death in older patients with diabetes [26,27]. Therefore, lower consumption of proteins may have accelerated the mortality risk for frail patients with diabetes aged ≥ 75 years. Therefore, it would be of great importance for these patients to consume sufficient proteins, as well as total energy, from the viewpoint of survival. In contrast, younger patients with diabetes would benefit from sufficient total energy intake, rather than protein intake, to reduce mortality.

The result that patients with a protein intake of 1.15–1.41 g/kg body weight (BW) had the lowest risk of mortality, especially those aged > 75 years with diabetes, is consistent with the recommendation from the European Society for Nutrition and Metabolism [28], that is, protein intake of at least 1.0–1.2 g/kg BW is necessary for older people to maintain the mass and quality of muscles, and protein intake of 1.2–1.5 g/kg BW is needed for patients with acute or chronic disease at high risk of malnutrition.

The finding that decreased intake of vegetable protein is associated with increased mortality was particularly remarkable in this study, but not the excess intake of animal protein. Reports on the effects of animal and vegetable protein intake on mortality are inconsistent in the general population [29,30]. Our results are consistent with the report that good survival rates were observed among patients aged > 75 years with diabetes and with healthy eating patterns, including foods rich in fish and vegetables, but not in those aged 65–74 years in the J-EDIT study [31]. In contrast, the JDCS study, including relatively young patients with diabetes, reported that high meat intake was associated with an elevated incidence of CHD [32]. Therefore, the effect of animal or vegetable protein intake on adverse outcomes may vary with the ages of patients with diabetes.

The relationship between protein intake and death may be influenced by carbohydrate intake, total energy intake, renal function, and food culture. In the group with low energy intake (<1533 kcal), mortality risk in the group with the highest protein intake was higher than that in the Q3 group, although it was not significant. The group with low energy and high protein intakes seems to be on a low carbohydrate diet. Replacing carbohydrate intake with animal protein intake could result in increased mortality and cardiovascular mortality in patients with type 2 diabetes, as well as in the general population [33,34]. The balance between animal protein and carbohydrate intakes may be important for survival in patients with diabetes.

In the present study, the changes in eGFR were not significantly different among the quartile groups of protein intake. The evidence regarding the effects of protein intake on renal function has been inconsistent [35,36,37]. In our study, patients’ renal function was maintained, and the mean protein intake in the highest group was 1.7 ± 0.3 g/day/kg BW. Our results suggest that with good renal function and microalbuminuria, as in our study, increased protein intake may not have adversely affected renal function in diabetic patients.

This study’s strengths lie in the follow-up of 2494 patients with diabetes, including the elderly, over a long period of 6–8 years. The study patients were treated by a doctor who specializes in diabetes, received dietary guidance and medication, and did not include untreated diabetic individuals. The association between low vegetable protein intake and mortality was observed in diabetic patients aged ≥ 75 years after adjusting covariates.

Several limitations of this study deserve mention. First, causality was unclear because this study was a longitudinal study. Second, we investigated the cause of death, but given the limited number of cases, analysis of causes of death, such as cardiovascular disease and cancer, has not been completed. Third, our study is a pooled analysis of two study populations. However, this analysis appears to be valid because the methods of nutritional assessment and outcomes of the two studies were quite similar [38]. Fourth, unmeasured confounding factors, such as sarcopenia, frailty, and socioeconomic status, and the uncertainty of physical activity evaluation by a self-administered questionnaire may have affected the association between protein intake and death.

## 5. Conclusions

Low protein intake was associated with higher mortality in patients with type 2 diabetes in the pooled analysis of two Japanese cohorts. The association between protein intake and mortality in those aged ≥ 75 years remained, after adjusting for covariates. Nutritional support concerning protein intake should vary with age. Adequate protein intake may be necessary in patients aged ≥ 75 years with diabetes. We think it is worth considering nutritional education, modification of the diet, ways to add foods to increase protein intake, and the use of supplementation for these older patients. On the other hand, whole optimal total energy intake, as well as protein intake, is required in younger patients with type 2 diabetes. Further interventional studies are necessary to determine whether protein supplementation can reduce mortality in patients aged ≥ 75 years with diabetes mellitus.

## Figures and Tables

**Table 1:** Baseline clinical characteristics of 2494 patients with type 2 diabetes according to quartiles of protein intake.

	Q1 (<0.92 g/kg BW) (*n* = 624)	Q2 (0.92–1.15 g/kg BW) (*n* = 623)	Q3 (1.15–1.41 g/kg BW) (*n* = 624)	Q4 (>1.41 g/kg BW) (*n* = 623)	*p*–Value
Age (years)	63.2 ± 9.0	63.1 ± 9.2	63.7 ± 8.8	63.8 ± 8.3	0.38
Women (%)	38.3	50.9	51.9	58.9	<0.01
HbA1c (%)	7.9 ± 1.1	7.9 ± 1.1	8.0 ± 1.2	8.0 ± 1.2	0.36
Duration of diabetes (years) (*n* = 1899)	10.6 ± 7.1	11 ± 6.9	11.1 ± 7.6	12.1 ± 7.7	0.01
Body mass index (kg/m^2^) (*n* = 2481)	24.9 ± 3.2	23.7 ± 3.0	22.9 ± 2.8	21.7 ± 2.8	<0.01
Systolic blood pressure (mmHg) (*n* = 2482)	134.6 ± 15.4	134.5 ± 16.6	132.8 ± 15.9	132.0 ± 16.9	<0.01
LDL cholesterol (mg/dL) (*n* = 2438)	121.2 ± 32	125.7 ± 31.3	120.8 ± 33.4	119.0 ± 30.4	<0.01
Ex- or current smoker (%) (*n* = 2350)	29.8	26.1	18.4	19.8	<0.01
Alcohol intake (%) (*n* = 2361)	39.3	37.3	34.0	30.7	0.01
Exercise (%) (*n* = 2362)	55.9	59.9	61.5	60.4	0.22
Urine albumin creatinine ratio * (mg/g Cr) (*n* = 2342)	23.0(10.7–68.3)	21.2 (9.8–65.1)	20.1 (9.7–58.6)	19.0 (9.4–53.3)	0.17 **
eGFR (mL/min/1.73 m^2^) (*n* = 2473)	78.0 ± 31.0	79.4 ± 28.1	80.6 ± 29.0	80.2 ± 25.2	0.40
History of cardiovascular disease (*n* = 2480)	41/621 (6.6%)	44/623 (7.1%)	34/619 (5.5%)	34/617 (5.5%)	0.57
History of stroke (*n* = 2479)	38/621 (6.1%)	31/622 (5.0%)	25/619 (4.0%)	28/617 (4.5%)	0.37
Protein intake (g/day/kg BW)	0.8 ± 0.1	1.0 ± 0.1	1.3 ± 0.1	1.7 ± 0.3	<0.01
Protein energy ratio (%)	13.8 ± 1.8	15.1 ± 1.9	16.1 ± 1.8	17.5 ± 2.2	<0.01
Carbohydrate intake (g/day/kg BW)	3.3 ± 0.7	3.8 ± 0.7	4.4 ± 0.8	5.0 ± 1.0	<0.01
Carbohydrate energy ratio (%)	58.4 ± 6.7	55.6 ± 6.0	54.2 ± 5.4	51.1 ± 6.0	<0.01
Fat intake (g/day/kg BW)	0.6 ± 0.2	0.8 ± 0.2	1.0 ± 0.2	1.3 ± 0.3	<0.01
Fat energy ratio (%)	24.1 ± 5.1	26.2 ± 4.5	27.5 ± 4.3	29.4 ± 4.7	<0.01
Total energy intake (kcal/day)	1410.9 ± 258.2	1622.0 ± 287.5	1818.8 ± 306.3	2071.6 ± 384.5	<0.01
Total energy intake (g/day/kg BW)	22.3 ± 3.6	27.5 ± 3.5	32.0 ± 3.9	39.5 ± 6.6	<0.01

Data are mean ± SD or *n* (%). * Median (Interquartile range) is shown; ** *p*-value for Kruskal-Wallis one-way analysis of variance. BW, body weight; HbA1c, hemoglobin A1c; Cr, creatinine.

**Table 2:** Cox regression analysis of quartiles of protein intake and all-cause mortality.

	n*	Q1 (<0.92 g/kg BW)		Q2 (0.92–1.15 g/kg BW)		Q3 (1.15–1.41 g/kg BW)		Q4 (>1.41 g/kg BW)	*p* for Trend
		HR (95% CI)	*p*	HR (95% CI)	*p*	HR (95% CI)	*p*	Reference	
Model 1	152	1.83 (1.17–2.84)	**0.008**	1.28 (0.80–2.05)	0.302	0.89 (0.53–1.49)	0.654	1	**0.002**
Model 2	142	1.95 (1.18–3.21)	**0.009**	1.43 (0.87–2.36)	0.155	0.79 (0.46–1.37)	0.405	1	**0.001**
Model 3	135	2.26 (1.34–3.82)	**0.002**	1.73 (1.03–2.91)	**0.038**	0.92 (0.52–1.64)	0.785	1	**<0.001**
Model 4	135	1.93 (0.87–4.26)	0.106	1.56 (0.82–2.98)	0.177	0.87 (0.47–1.61)	0.665	1	**0.047**

*n* *: event number; model 1: crude model; model 2: adjusted for age, sex, BMI, HbA1c, SBP, LDL cholesterol, smoking, and alcohol intake; model 3: model 2 + UACR, eGFR, and exercise; model 4: model + energy intake. BW, body weight; CI, confidence interval; eGFR, estimated glomerular filtration rate; HbA1c, hemoglobin A1c; HR, hazard ratio; SBP, systolic blood pressure; UACR, urine albumin creatinine ratio. Bold values denote statistical significance at the *p* < 0.05 level.

**Table 3:** Subgroup analyses of association between quartiles of protein intake and all-cause mortality in patients with type 2 diabetes.

		Q1 (<0.92 g/kg BW)		Q2 (0.92–1.15 g/kg BW)		Q3 (1.15–1.41 g/kg BW)		Q4 (>1.41 g/kg BW)	*p* for Trend
	*n* *	HR (95% CI)	*p*	HR (95% CI)	*p*	HR (95% CI)	*p*	Reference	
**Men**									
Model 1	98	1.75 (0.98–3.13)	0.058	1.31 (0.7–2.45)	0.399	0.93 (0.47–1.84)	0.834	1	**0.018**
Model 2	91	2.04 (1.07–3.86)	**0.029**	1.43 (0.74–2.77)	0.290	0.79 (0.38–1.65)	0.539	1	**0.005**
Model 3	87	2.19 (1.12–4.28)	**0.021**	1.61 (0.82–3.17)	0.170	0.92 (0.43–1.93)	0.817	1	**0.005**
Model 4	87	1.43 (0.54–3.77)	0.475	1.22 (0.54–2.75)	0.634	0.8 (0.37–1.75)	0.582	1	0.335
**Women**									
Model 1	54	1.51 (0.73–3.12)	0.271	1.14 (0.56–2.34)	0.712	0.77 (0.35–1.72)	0.526	1	0.209
Model 2	51	1.88 (0.81–4.37)	0.143	1.59 (0.74–3.43)	0.235	0.85 (0.36–1.98)	0.702	1	0.081
Model 3	48	2.35 (0.99–5.58)	0.053	2.13 (0.95–4.77)	0.068	1.00 (0.40–2.48)	0.993	1	**0.021**
Model 4	48	4.22 (1.04–17.16)	**0.044**	**3.15 (1.05–9.47)**	**0.041**	1.29 (0.45–3.64)	0.636	1	**0.019**
**Age ≥ 75 yrs**									
Model 1	27	2.51 (0.86–7.34)	0.094	1.87 (0.63–5.57)	0.263	0.48 (0.11–2.00)	0.311	1	**0.017**
Model 2	27	4.38 (1.22–15.8)	**0.024**	2.16 (0.66–7.09)	0.206	0.62 (0.14–2.70)	0.521	1	**0.007**
Model 3	25	5.59 (1.45–21.55)	**0.012**	2.63 (0.71–9.84)	0.150	0.83 (0.17–3.93)	0.810	1	**0.004**
Model 4	25	15.3 (2.17–107.3)	**0.006**	**5.45 (1.03–28.76)**	**0.046**	1.05 (0.21–5.23)	0.950	1	**0.004**
**Age: 65–74 yrs**									
Model 1	71	1.33 (0.73–2.42)	0.347	0.85 (0.43–1.7)	0.649	0.71 (0.35–1.43)	0.336	1	0.261
Model 2	62	1.33 (0.66–2.69)	0.427	0.84 (0.4–1.79)	0.654	0.57 (0.26–1.26)	0.163	1	0.280
Model 3	59	1.27 (0.62–2.64)	0.513	0.88 (0.41–1.89)	0.750	0.52 (0.23–1.19)	0.122	1	0.333
Model 4	59	1.03 (0.31–3.38)	0.962	0.77 (0.29–2.04)	0.598	0.48 (0.2–1.18)	0.111	1	0.720
**Age < 65 yrs**									
Model 1	46	2.67 (1.04–6.89)	**0.042**	2.14 (0.82–5.56)	0.120	1.93 (0.71–5.21)	0.196	1	**0.044**
Model 2	45	2.67 (0.98–7.30)	0.055	2.36 (0.88–6.32)	0.087	1.80 (0.65–5.03)	0.259	1	**0.048**
Model 3	44	3.33 (1.11–10.02)	**0.032**	2.86 (0.96–8.52)	0.060	2.31 (0.75–7.10)	0.144	1	**0.033**
Model 4	44	1.32 (0.29–5.93)	0.715	1.53 (0.42–5.55)	0.518	1.71 (0.53–5.50)	0.372	1	0.924
**HbA1c ≥ 7.5%**									
Model 1	105	1.72 (1.03–2.87)	**0.039**	1.28 (0.75–2.18)	0.364	0.54 (0.27–1.06)	0.071	1	**0.004**
Model 2	100	1.70 (0.95–3.04)	0.073	1.42 (0.81–2.49)	0.226	0.52 (0.26–1.06)	0.071	1	**0.011**
Model 3	96	1.99 (1.08–3.66)	**0.027**	1.65 (0.92–2.98)	0.095	0.64 (0.31–1.32)	0.227	1	**0.004**
Model 4	96	1.79 (0.68–4.74)	0.241	1.55 (0.72–3.31)	0.261	0.62 (0.29–1.33)	0.220	1	0.099
**HbA1c < 7.5%**									
Model 1	47	2.33 (0.97–5.62)	0.060	1.28 (0.48–3.43)	0.625	2.15 (0.87–5.32)	0.099	1	0.147
Model 2	42	2.86 (1.03–7.91)	**0.043**	1.51 (0.52–4.35)	0.446	1.90 (0.69–5.25)	0.213	1	0.067
Model 3	39	3.26 (1.09–9.78)	**0.035**	1.85 (0.60–5.7)	0.286	1.94 (0.64–5.91)	0.241	1	**0.040**
Model 4	39	2.44 (0.55–10.73)	0.239	1.51 (0.41–5.64)	0.539	1.76 (0.55–5.62)	0.342	1	0.303
**SBP ≥ 135mmHg**									
Model 1	69	2.22 (1.16–4.25)	**0.017**	1.05 (0.50–2.21)	0.890	0.79 (0.36–1.77)	0.574	1	**0.005**
Model 2	65	4.00 (1.85–8.67)	**<0.001**	1.51 (0.68–3.35)	0.311	0.86 (0.36–2.09)	0.746	1	**<0.001**
Model 3	62	5.04 (2.30–11.04)	**<0.001**	1.90 (0.84–4.30)	0.126	0.94 (0.37–2.38)	0.895	1	**<0.001**
Model 4	62	3.00 (0.89–10.09)	0.076	1.33 (0.48–3.73)	0.583	0.79 (0.30–2.11)	0.642	1	**0.039**
**SBP < 135 mmHg**									
Model 1	83	1.49 (0.81–2.74)	0.202	1.46 (0.8–2.67)	0.222	0.96 (0.49–1.88)	0.904	1	0.103
Model 2	77	1.13 (0.57–2.25)	0.732	1.39 (0.73–2.65)	0.311	0.79 (0.38–1.60)	0.507	1	0.414
Model 3	73	1.17 (0.56–2.44)	0.669	1.55 (0.79–3.04)	0.203	0.92 (0.44–1.92)	0.821	1	0.408
Model 4	73	1.14 (0.40–3.30)	0.803	1.52 (0.66–3.52)	0.324	0.91 (0.42–1.99)	0.814	1	0.583
**Total energy intake < 1533 kcal/day**									
Model 1	71	1.53 (0.37–6.32)	0.554	0.88 (0.2–3.84)	0.867	0.93 (0.19–4.46)	0.924	1	0.080
Model 2	66	1.03 (0.22–4.85)	0.969	0.80 (0.17–3.72)	0.777	0.68 (0.13–3.57)	0.648	1	0.452
Model 3	63	0.74 (0.16–3.52)	0.707	0.68 (0.15–3.21)	0.629	0.58 (0.11–3.06)	0.519	1	0.840
**Total energy intake: 1533–1833 kcal/day**									
Model 1	41	1.25 (0.44–3.55)	0.680	1.60 (0.66–3.86)	0.295	1.02 (0.39–2.68)	0.969	1	0.388
Model 2	39	1.37 (0.33–5.73)	0.671	1.55 (0.53–4.52)	0.418	0.91 (0.32–2.55)	0.850	1	0.425
Model 3	37	1.82 (0.42–8.01)	0.425	1.96 (0.64–6.07)	0.240	1.04 (0.34–3.15)	0.943	1	0.246
**Total energy intake ≥ 1834 kcal/day**									
Model 1	40	0.57 (0.08–4.26)	0.587	1.23 (0.52–2.87)	0.640	0.74 (0.35–1.56)	0.427	1	0.839
Model 2	37	0.91 (0.11–7.58)	0.930	1.30 (0.48–3.52)	0.599	0.66 (0.29–1.52)	0.329	1	0.936
Model 3	35	1.01 (0.12–8.54)	0.996	1.48 (0.54–4.06)	0.451	0.78 (0.34–1.83)	0.572	1	0.698
**eGFR** **≥ 75.4 mL/min/1.73 m^2^**									
Model 1	54	2.72 (1.25–5.94)	**0.012**	1.47 (0.62–3.50)	0.379	1.42 (0.60–3.37)	0.426	1	**0.010**
Model 2	51	2.76 (1.19–6.42)	**0.018**	1.60 (0.66–3.88)	0.295	1.26 (0.51–3.12)	0.620	1	**0.013**
Model 3a	48	4.32 (1.60–11.64)	**0.004**	2.57 (0.92–7.21)	0.072	2.14 (0.75–6.12)	0.155	1	**0.003**
Model 4a	48	2.20 (0.53–9.06)	0.276	1.66 (0.49–5.62)	0.418	1.73 (0.58–5.18)	0.330	1	0.341
**eGFR < 75.4 mL/min/1.73 m^2^**									
Model 1	98	1.38 (0.81–2.37)	0.239	1.13 (0.65–1.98)	0.665	0.66 (0.34–1.28)	0.217	1	0.085
Model 2	91	1.58 (0.84–2.96)	0.153	1.35 (0.74–2.47)	0.334	0.61 (0.30–1.24)	0.174	1	**0.039**
Model 3a	87	1.68 (0.88–3.22)	0.115	1.50 (0.81–2.78)	0.192	0.61 (0.30–1.27)	0.188	1	**0.025**
Model 4a	87	1.92 (0.72–5.15)	0.196	1.64 (0.75–3.58)	0.217	0.64 (0.30–1.39)	0.261	1	0.075
**Duration of diabetes ≥ 9.8 years**									
Model 1	51	1.31 (0.64–2.68)	0.462	0.72 (0.32–1.62)	0.424	0.90 (0.41–1.99)	0.803	1	0.596
Model 2	47	2.00 (0.89–4.50)	0.093	1.05 (0.45–2.47)	0.909	0.78 (0.32–1.91)	0.589	1	0.088
Model 3	46	2.44 (1.04–5.69)	**0.040**	1.34 (0.54–3.28)	0.526	1.02 (0.40–2.55)	0.974	1	**0.036**
Model 4	46	1.88 (0.49–7.17)	0.357	1.13 (0.36–3.47)	0.837	0.93 (0.35–2.49)	0.891	1	0.354
**Duration of diabetes < 9.8 years**									
Model 1	47	3.20 (1.28–8.01)	**0.013**	2.21 (0.84–5.81)	0.108	1.52 (0.54–4.26)	0.430	1	**0.006**
Model 2	46	2.31 (0.87–6.15)	0.093	1.87 (0.69–5.05)	0.216	1.39 (0.48–3.97)	0.543	1	0.065
Model 3	44	1.93 (0.70–5.34)	0.204	1.80 (0.66–4.93)	0.250	1.17 (0.40–3.43)	0.772	1	0.123
Model 4	44	1.16 (0.26–5.28)	0.846	1.33 (0.40–4.45)	0.647	0.98 (0.31–3.07)	0.969	1	0.731

*n* *: event number; model 1: crude model; model 2: adjusted for age, sex, body mass index, HbA1c, SBP, LDL cholesterol, smoking, and alcohol intake; model 3: model 2 + UACR, eGFR, and exercise; model 3a: model 2 + UACR and exercise; model 4: model 3 + energy intake; model 4a: model 3a + energy intake. In subgroup analyses of age, sex, HbA1c, or eGFR, the variables were excluded, respectively. Bold values denote statistical significance at the *p* < 0.05 level.

**Table 4:** Association between animal or vegetable protein and all-cause mortality in patients with type 2 diabetes.

		Q1		Q2		Q3		Q4	*p* for Trend
	*n* *	HR(95% CI)	*p*	HR (95% CI)	*p*	HR (95% CI)	*p*	HR	
**Animal Protein**									
		<4.2 g/kg BW		4.2–5.8 g/kg BW		5.8–7.4 g/kg BW		>7.4 g/kg BW	
Model 1	149	1.70 (1.10–2.64)	**0.017**	1.18 (0.74–1.88)	0.494	0.87 (0.52–1.43)	0.573	1	**0.006**
Model 2	139	1.58 (0.96–2.59)	0.070	1.18 (0.71–1.96)	0.517	0.87 (0.51–1.47)	0.603	1	**0.030**
Model 3	132	1.69 (1.01–2.83)	**0.045**	1.34 (0.79–2.27)	0.271	0.99 (0.57–1.71)	0.965	1	**0.022**
Model 4	132	1.21 (0.66–2.22)	0.531	1.05 (0.59–1.86)	0.869	0.85 (0.48–1.50)	0.575	1	0.359
**Vegetable Protein**									
		<4.2 g/kg BW		4.2–6.1 g/kg BW		6.1–8.4 g/kg BW		>8.4 g/kg BW	
Model 1	150	2.32 (1.43–3.78)	**0.001**	1.85 (1.12–3.06)	**0.016**	1.43 (0.84–2.42)	0.184	1	**<0.001**
Model 2	140	2.21 (1.30–3.75)	**0.003**	1.77 (1.03–3.02)	**0.037**	1.35 (0.78–2.35)	0.286	1	**0.002**
Model 3	133	2.32 (1.35–3.98)	**0.002**	1.88 (1.09–3.25)	**0.024**	1.27 (0.72–2.26)	0.412	1	**0.001**
Model 4	133	1.92 (1.07–3.43)	**0.028**	1.65 (0.94–2.91)	0.082	1.22 (0.68–2.17)	0.501	1	**0.017**

*n* *: event number; model 1: crude model; model 2: adjusted for age, sex, body mass index, HbA1c, SBP, LDL cholesterol, smoking, and alcohol intake; model 3: model 2 +UACR, eGFR, and exercise; model 4: model 3+ total energy intake. Bold values denote statistical significance at the *p* < 0.05 level.
